# Workplace Violence toward Physicians and Nurses: Prevalence and Correlates in Macau

**DOI:** 10.3390/ijerph14080879

**Published:** 2017-08-04

**Authors:** Teris Cheung, Paul H. Lee, Paul S. F. Yip

**Affiliations:** 1 School of Nursing, Hong Kong Polytechnic University, Hong Kong, China; teris.cheung@polyu.edu.hk; 2 Centre for Suicide Research and Prevention, University of Hong Kong, Hong Kong, China; sfpyip@hku.hk

**Keywords:** doctors, nurses, workplace violence, occupational stress, mental health, Macau

## Abstract

This paper sets out to estimate the prevalence of workplace violence in relation to socio-demographic characteristics of physicians and nurses working in healthcare settings in Macau. *Background*: Concerted efforts worldwide to reduce workplace violence (WPV) have not yet removed medical-related professionals from the threat of patients’, family members’, and colleagues’ physical and other assaults in Southeast Asia. *Methods*: The study employs a cross-sectional design to estimate the prevalence and examines the socio-economic and psychological correlates of WPV among medical doctors and nurses in Macau. The data collection period spanned from August to December 2014. Multiple logistic regression examines the prevalence rates of WPV and its associated factors in doctors and nurses. *Results*: A total of 107 doctors (14.9%) and 613 nurses (85.1%) participated in the study; 57.2% had suffered WPV in the preceding year. The most common forms of workplace violence were verbal abuse (53.4%), physical assault (16.1%), bullying/harassment (14.2%), sexual harassment (4.6%), and racial harassment (2.6%). Most violence was perpetrated by patients and their relatives, colleagues, and supervisors. *Conclusions:* WPV remains a significant concern in healthcare settings in Macau. Macau’s local health authority should consider putting in place a raft of zero-tolerance policies designed to prevent it.

## 1. Introduction

The National Institute for Occupational Safety and Health (NIOSH) defines WPV workplace violence as “violent acts (including physical assaults and threats of assaults) directed toward persons at work or on duty” [1]. In 2014, the American Nurses Association further included lateral violence (acts between colleagues, bullying, hostility, abuse of authority, and sexual harassment) in the definition of WPV [2]. The last decade has seen a number of studies from around the world of the workplace violence (WPV) of patients or visitors in healthcare settings. Public health authorities, for their part, have attested to significant concern at non-fatal violence aimed at healthcare workers. The year 2013 saw a reported 25,630 incidents of WPV in the US, of which 74% apparently occurred in healthcare settings [3]. Workplace violence committed against medical occupational groups represented 10.2% of all workplace violence incidents. Research has consistently reported that violence incidence rates in public healthcare settings are higher than in the private health sector [3] Workplace violence remains a threat in healthcare setting across America [3]. Using the WPV instrument [4], Mai and colleagues [5] measured WPV among physicians and nurses (*n* = 672) in Macau. Mai’s study reported the prevalence of verbal abuse (56.8%), physical assault (13%), bullying/mobbing (32.1%), and sexual harassment (8.2%). Mai’s study, however; did not examine the prevalence rates of WPV for the two different professions. Yao [6] examined the impact of WPV on 758 physicians’ self-efficacy and job satisfaction in nine hospitals in Zhengzhou, Henan Province, China and reported that 63.2% of physicians experienced WPV in the past 12 months. A recent published cross-sectional survey in Hong Kong by Cheung and Yip [7] examined the prevalence and correlates of WPV towards 850 nurses; 44.6% had experienced WPV in the preceding year. Male nurses reported more WPV than their female counterparts. The most common forms of WPV were verbal abuse/bullying (39.2%), then physical assault (22.7%), and sexual harassment (1.1%). The most common perpetrators of WPV were patients (36.6%) and their relatives (17.5%), followed by colleagues (7.7%) and supervisors (6.3%) [7]. Cheung and Yip’s findings were also in line with other international studies [8,9,10]. Younger age, clinical inexperience, shift work, and anxiety increased the risk of verbal abuse [7,11]. The key perpetrators are mostly patients and their relatives [7,10,12]. Colleagues and supervisors have also been identified as committing WPV [11,13]. It seems natural to suppose that occupational groups, physicians [14,15,16], and nurses [15,16,17,18,19] stand at a disproportionately high risk of WPV. Nevertheless, existing research tends to focus on specific professions (that is, only potentially one of these two jobs), recording their experience in cross-sectional designs. WPV can be taken to arise out of poor communication between co-workers and patients [20], a failure to observe patients vigilantly, settings’ non-compliance with WPV prevention policies, and inadequate assessment of patients’ aggravators and other behaviors [21]. To date, very little research compares prevalence estimates for WPV for different medical-related professionals. To the author’s knowledge, no study has as yet measured the prevalence and correlates of WPV towards physicians and nurses in Macau—a gap filled by the present work. We also aimed to examine whether there is a statistical difference in the prevalence estimates of WPV towards physicians and nurses in this study.

## 2. Materials and Methods

This paper sets out to estimate the prevalence of workplace violence in relation to certain socio-demographic characteristics of the physicians and nurses working in healthcare settings in Macau. Details of the study has been published elsewhere [5]. In brief, this study used a cross-sectional design. Convenience sampling was sourced from six health centers supervised by the Macau Health Bureau, namely, Coloane Health Center, Coloane Shek Pai Wan Temporary Health Center, Taipa Elderly Macau University of Science and Technology Hospital, the Macau Sino-Portuguese Nurses Association, the Macau Association of Medical Volunteers, and the Macau Surgical Association. As of 30 June 2014, Macau’s population stood at 636,200 (51% of whom were female) [22]. In 2014, the population was served by 3582 healthcare professionals (1592 physicians and 1990 nurses) working in the public or private health sector. Healthcare workers, both male and female, who had worked for these organizations for at least a year were invited to participate in this study.

The study used the “Workplace Violence in the Health Sector Country Case Studies Research Instruments Survey Questionnaire” as circulated by the International Labor Office (ILO), International Council of Nurses (ICN), Public Services International (PSI), and WHO [4]. This questionnaire comprises five domains: (1) gathers socio-demographic information (e.g., the gender, age and marital status of respondents); (2) notes physical assaults, capturing the perpetrators of abuse, its apparent motivations, and management; (3) pertains to psychological abuse, such as verbal abuse, bullying, or harassment; and (4) concerns health policy and canvasses suggestions on the prevention of WPV. Nine hundred questionnaires were distributed. The data collection period spanned August to December 2014. In this paper, the definition of WPV incorporated the original definition framed by the World Health Organization in the box below:
Verbal abuse—vulgarity, insults, sniggering Bullying—unreasonable workloads or shifts Physical abuse—physical assault, slapping, kicking, other forms of physical affront Sexual harassment—verbal remarks of a sexual nature, lewd gestures or hints, any form of sexualized physical action

## 3. Results

### 3.1. Socio-Demographic, Clinical, and Other Characteristics of the Sample Population

A total of 720 healthcare professionals were recruited to the study (14.9% physicians and 85.1% nurses); 56.3% were married and 40.7% single. The majority of respondents (78.8%) were female and worked on a shift rotation (75.6%). Respondents were aged between 20 and 60 or above, with nearly 60% falling in an age range between 25 and 39 years old. The mean age was between 25 and 34 (SD: 1.07). 62.4% of respondents had ≤10 years of clinical experience. The study’s response rate was 80%. Table 1 reports the frequency distribution of respondents by socio-demographic characteristics and selected variables.

Whether or not participants had encountered different types of workplace violence (WPV) were coded as a dichotomous response (yes/no). The prevalence of WPV was examined and presented in terms of frequency and the proportion of those encountering it (%). Prevalence estimates (%) were presented at 95% confidence intervals (CI) calculated from the SE. Binary and multivariate logistic regression analyses measured the strength of associations between variables and sought to identify significant predictors for the outcome variable, WPV. All tests were two-tailed, with the level of significance set as *p* < 0.05. Results were presented as odds ratios (ORs) and 95% CIs.

All independent variables with a *p* value of <0.25 in the bivariate analysis were taken by the study as important risk factors for physical assault, verbal abuse, bullying, and sexual and racial harassment and entered into multivariate logistic regression. Our choice of a cutoff point (*p* < 0.25) for selecting potentially influential variables was based on Hosmer and Lemeshow’s [23] recommendations to avoid leaving out potentially important covariates that had not yet emerged as significant in univariate analysis. At the same time, this cutoff was used to screen out variables of questionable importance. A forward likelihood ratio (LR) was used to identify variables that could be plausibly associated with physical assault, verbal abuse, bullying, and sexual and racial harassment in five separate models.

### 3.2. Prevalence of WPV

Overall, 57.2% of respondents reported some form (physical and psychological) of WPV in the preceding year. Data showed a prevalence of 16.1% for physical assault, 53.4% for verbal abuse, 14.2% for bullying/harassment, 4.6% for sexual harassment, and 2.6% for racial harassment. Nurses were at a significantly higher risk of physical assault and verbal abuse as against physicians (Table 2).

Bullying, sexual harassment, and racial harassment, however, showed no significant difference in prevalence estimates between the medical and nursing groups. With the exception of sexual harassment, the most common perpetrators of all kinds of WPV were patients, patients’ relatives, and professionals’ colleagues/supervisors. Patients were the main perpetrators for all kinds of WPV, followed by patient’s family members and colleagues (Table 3). We also noted that there were statistically significant differences of physical assault, perpetrated by patients and his/her family members towards physicians and nurses (χ^2^ = 15.30 and 16.77, respectively; both *p*s < 0.05). Physicians also did not report any verbal abuse from peer colleagues, compared with nurses (χ^2^ = 4.46, *p* < 0.05) (Table 3).

### 3.3. Binary Logistic Regression Analysis

Results showed that being married/cohabitant and single emerged as a significant correlate of physical abuse and racial harassment, respectively. Four significant variables were closely associated with physical abuse and they were (1) nurses; (2) less than 10 years of work experience; (3) public sector; (4) shift work; and (5) night duty. Direct patient care of those vulnerable patient groups (i.e., newborn, infants, children, elderly, physical disability, intellectual disability, maternity, psychiatric disorder, HIV, terminal illness) was strongly associated with all types of WPV. Worry about WPV also emerged as a significant correlate of physical assault, verbal abuse, bullying, and sexual harassment (Table 4).

### 3.4. Multivariate Logistic Regression

In the final model, six variables—shift duty, directly caring for children, directly caring for the elderly, spending more than half of the time directly caring for patients with physical disabilities and psychiatric patients, and worrying about WPV—emerged as significant correlates of physical assault (Table 5). The strongest correlate was directly caring for elderly, which had an adjusted odds ratio (aOR) of 5.61 times, followed by worrying about WPV (at an aOR 4.51) and shift duty (an aOR 2.48). Participants with 50% of time devoted to direct patient care (with physical disabilities and psychiatric disorders) were 1.7 times and 1.9 times more likely to report physical assault than those without direct patient care.

For verbal abuse, six variables—(1) <10 years of work experience; (2) night duty; offering direct patient care, to (3) elderly; (4) 50% direct patient care of psychiatric patients and (5) maternity patients; and 6) worrying about WPV—emerged as significant correlates of verbal abuse (Table 5). The strongest correlate was worry about WPV (aOR 3.90), followed by 50% of directly offering care (to psychiatric patients) (aOR 3.22) and night duty (at an aOR of 1.93). Participants who worry about WPV were nearly four times more likely to report verbal abuse than those who do not. Participants directly caring for elderly and maternity patients were 1.6 and 0.6 times, respectively, more likely to experience verbal abuse than those not charged with care in these units. Participants on night duty were 1.9 times more likely to be verbally abused than staff on regular duty. Participants with less than 10 years of work experience were 1.5 times more likely to report verbal abuse than those with >11–20 years of work experience.

Six variables—(1) shift duty; and direct patient to (2) children; (3) elderly; devoted 50% of direct patient care to (4) physical disability; (5) psychiatric disorder; and (6) their worrying about WPV—emerged as significant correlates of bullying (Table 5). Direct care (elderly) was the strongest correlate of WPV (aOR 5.61), followed by worrying about WPV (aOR 4.51) and shift duty (aOR 2.48). Participants who worked in psychiatric units and physical disability units were 1.9 times and 1.7 times more likely than those in general units to experience bullying at work.

For sexual harassment, three variables emerged as significant correlates. The strongest correlate was directly caring for patients (aOR 4.58), followed by offering home care (aOR 2.84), and directly caring for psychiatric patients (aOR 2.69) (Table 5).

Two variables—devoting 50% direct patient care in physical disability units and direct patient care for infants—emerged as significant correlates of racial harassment (Table 5). Participants requiring 50% direct patient care for physical disability patients were four times more likely than those without direct patient care to report racial harassment. Participants with direct patient care for infants were 2.8 times more likely to experience racial harassment than those without.

## 4. Discussion

### 4.1. WPV Prevalence Comparison between Hong Kong, China, United States, and United Kingdom

Our overall prevalence estimates for workplace violence and harassment for medical-related professionals came in at 57.2% which was even higher than in a recent (2013) cross-sectional study in Hong Kong of 850 nurses, which revealed an overall prevalence rate of WPV of 44.6% [7]. However, alongside a similar prevalence study of Hong Kong nurses (*n* = 420, 76% verbal abuse, 45% bullying, 18% physical assault, 12% sexual harassment) conducted a decade ago, the prevalence rate of WPV in this study seems lower. Our results confirmed the findings of other researchers’ work in suggesting that verbal abuse, physical assault and bullying were the most common forms of WPV [24,25,26]. One noteworthy result of the study was the relatively low prevalence of sexual harassment and racial harassment, although even these rates of incidence are alarming in that prevalence studies may have the risk of under-reporting and recall bias [27]. More significantly, some respondents may consider the risk of WPV as part of their job [21,28,29] and may not report it for fear of retaliation [25], fear of blame, excessive paperwork, and to go through a time-consuming reporting process [30].

A cross-sectional study investigating prevalence estimates for WPV towards physicians in China in 123 public hospitals in Shanghai, Hubei and Gansu Provinces (*n* = 1656 stratified random samples) [31] came up with an overall prevalence of over 90% of WPV (verbal abuse, 92.8%; threatened assaults, 88.1%; and actual physical assault, 81.0%). In another comparable case, a recent large-scale cross-sectional study of 6300 nurses in Minnesota found an incidence of physical assault by patients and visitors of 13.2 per 100 healthcare professionals (95% CI 12.2–14.3) in the past year [17,21]. This study used a nested-control design to compare 310 cases across 946 control subjects. The odds ratio for physical assault was found to go up in nursing homes (or long-term care facilities) (aOR 2.6, 95% CI: 1.9–3.6), emergency departments (aOR 4.2, 95% CI 1.3–12.8) and psychiatric departments (aOR 2.0, 95% CI 1.1–3.7). Our prevalence of physical assault was comparatively lower than Gerbeich et al.’s and Shi et al.’s findings [28,31].

Another cross-sectional study from the United States researched 762 registered female nurses between 26 and 64 and with more than 10 years of experience; 76.0% of the participants had experienced violence in the preceding year (54.2% physical abuse by patients; 3.5% physical abuse by visitors, and 29.9% verbal abuse by visitors) [25]. Our study findings on the prevalence of physical assault (87.1% physical assault by patients; 11.2% by visitors) again bore a close comparison to those of Speroni et al.’s.

Over a decade ago, Whittington and Shuttleworth [26] used a cross-sectional study to survey 396 nurses in a general hospital in England. Twenty-one percent reported physical assault and 43% reported verbal abuse in relation to WPV. There is a reason to suppose geographic differences in the prevalence of physical assault and verbal abuse across the globe, possibly due to measurement constructs of WPV and variations in normative behavior across cultural and ethnic contexts.

### 4.2. Occupational Group

Bivariate analysis showed that nurses reported more physical assaults and verbal abuse than physicians. Otherwise, differences between occupational groups did not appear as significant correlates in the final model in multivariate analysis. A recent longitudinal study in the United States also found a greater statistical likelihood of nurses being physically assaulted or threatened than physicians [15]. These findings would be expected, in that nurses’ ‘frontline’ position means they directly interact with patients and their relatives for longer periods of time and are proportionally at higher risk of insult and injury. Patients presented in healthcare settings for consultation and treatment, and so depend directly on physicians for their treatment needs. This may make them less likely to strike out at doctors.

### 4.3. Public Health Sector

Of particular interest, physicians and nurses working in public health sectors were more prone to report physical assault, verbal abuse, and bullying than those working in the private sector in the binary logistic regression analysis. The public health sector, however, did not turn up as a significant correlate in the multivariate logistic regression analysis. It is likely that staff-patient ratios in the public health sector are higher than in private healthcare. Depending on patients’ insurance packages, Macau citizens may have to pay relatively heavily for medical consultation and treatment in private hospitals; or, alternatively, patients may seek cheaper care in public hospitals, reconciling themselves to longer waiting times for diagnosis and follow-up care. Yet this will, of course, dispose some to anxiety and violent behavior. Overcrowding, staff shortages, and dissatisfaction with outcomes in both in-patient and out-patient units in the public health sector may lead to physical or non-physical forms of WPV. Other factors in the personal dynamics behind WPV getting out of hand may be power differences between medical-related personnel and patients, socio-cultural dynamics, and hospitals’ real and perceived inefficiencies [32]. A recent study (*n* = 4392) in Finland [33] reported that healthcare professionals’ physical environment may affect their psychological well-being. Those working in a run-down settings are more likely to report bullying. In Macau, also, it is plausible to think of hospitals’ physical environments as a precursor of WPV.

### 4.4. Shift Work

Results showed that participants on shift work rotation were at higher risk of exposure to physical assault, verbal abuse, and bullying than those on regular day duty. Clinical units tend to have lighter rosters during meal breaks and during duty handover, any slight delay in attending patients’ needs may provoke abuse or hectoring from service users or their relatives. Some healthcare units in the public sector suffer from shortages of nurses, causing patients to wait for a long time in a sometimes crowded, noisy, and unstable environment [17]. These features were possibly misinterpreted by patients as betraying a lack of care, or of concern for them, on the healthcare professionals’ part. The nurses reporting higher incidences of abuse tended to care for patients directly as primary providers over the day in different shifts. Other healthcare workers would have come into contact with patients in the course of their recovery and rehabilitation, or in therapy (e.g., physiotherapists, occupational therapists, dietitians, medical social workers), but these sessions were typically scheduled for shorter periods. It is possible patients could have perceived the second set of care providers as higher status, and so less fitting as targets of frustration or abuse; or that frustration could have built up in their minds with their regular principal care-providers. There may be a suggestion, then, that the nature of the care provided by professionals, its duration, their job titles, and the discontinuous manner in which holistic care is provided may all serve as precursors or predictors of WPV.

### 4.5. Direct Patient Care

Our results consistently showed at the multivariate stage of analysis that directly caring for patients seemed to be a significant correlate of WPV in different domains. Most occupational groups engaged in direct care stood a higher risk of violence than those who did not [34,35]. Directly caring for children, psychogeriatric patients, and maternity patients correlated significantly with professionals being victims of physical assault and verbal abuse. Direct care of infants correlated significantly with racial harassment. Interestingly, no previous empirical study has stated that working with children or in pediatric units could be a risk factor for WPV. One possibility is that these young perpetrators of violence may not be receptive to, or fully understand, the treatment process in terms of, e.g., the medication, analgesics, and injections administered by unfamiliar, possibly threatening, healthcare professionals. Children could then have been susceptible to tantrums or other behavioral manifestations in the clinical environment. Further investigation would be needed to check whether the degree of WPV suffered by professionals in pediatric units stemmed from any lack of training in their caring for sick children in Macau. In Hong Kong, working in child/pediatric units is considered a clinical specialty. Nurses enroll on a specialty training course in clinically managing children and adolescents as preparation for their being able to deal with patients’ behavioral problems and so improve treatment outcomes.

It was notable that working in emergency departments did not turn out to be a risk factor of WPV, partly due to the fact that less than 10% (*n* = 70, 9.7%) of respondents worked in that setting. This result should be treated with caution. The possibility cannot be ruled out that WPV is more prevalent in these units than in others, as might have emerged had more respondents from Accident and Emergency units participated in the survey. 

By contrast, 15.3% of the survey’s participants worked in maternity units, 22.1% in child units, 14.7% in psychiatric units, and the largest proportion (63.8%) in elderly units. Existing research has suggested that working in long-term care facilities, nursing homes, emergency departments, and psychiatric units correlated with a significantly higher risk of exposure to WPV [28,36]. Arguably, child, maternity, and psychogeriatric units all offer high levels of care to vulnerable patients around the clock. As the frontline care providers of these units, nurses must directly tend to patient care, often in close contact. In receiving care, patients may experience a loss of control and fail to follow the medical procedures to which they are being submitted. They may also be in pain and experience feelings of powerlessness. These feelings of frustration, pain and anger may be sharpened and directed towards healthcare workers, especially in situations where workers seek to limit patients’ disruptive or uncooperative behaviors (sometimes for the sake of ward management or safety). In these circumstances, patients and their family members may strike out at frontline staff in anger at the apparent (or real) denial of care, or through misunderstandings, in a spirit of distrust.

Study respondents working in maternity units, children units, psychogeriatric units, physical disability units, and psychiatric units reported verbal abuse with particular frequency. A total of 384 participants reported verbal abuse, 7.4% of whom (*n* = 53) could be considered immigrants, while less than one third (*n* = 17) had worked in Macau for over five years. Approximately 1 in 7 immigrant workers experienced verbal abuse in the year before the study. This abuse could also have arisen in connection with the victims’ lack of clinical experience in maternity units. Maternity unit patients would have been overwhelmingly local Macau citizens while immigrant professionals would have received their licenses to practice in different countries. The correlation between the length of clinical experience and occurrences of verbal abuse revealed the two factors were not independent (Fisher’s exact test: *p* = 0.031), showing that verbal abuse becomes less frequent as professionals gain experience.

Nonetheless, WPV was not absent among experienced clinicians or nurses contributing to the study, any more than it was in Privitera et al.’s [16] comparable study. It may be that tempers inevitably flare, and that some professionals feel they are being bullied, given variations between the standard of care expected by managers and that delivered by inexperienced staff. Cultural and ethnic tensions between care providers and recipients may also make verbal abuse inevitable. Expectant or young mothers tend to feel acute anxiety or somatic discomfort after childbirth, leading to their placing unrealistic demands on healthcare workers. Frontline maternity unit workers were, thus, at higher risk of being verbally abused or bullied.

With the exception of sexual harassment, working in psychiatric settings was found to be significantly correlated with all types of WPV. Nurses taking care of patients with mental illnesses or neurological problems were at higher risk of WPV [19,36,37]. It is plausible that these psychogeriatric units may have in-patients with dementia or Alzheimer disease. Due to cognitive deficits and impaired memory, these patients may become confused, agitated, and disoriented as to date, time, and place. Nurses may, thus, be at a disproportionate risk of the violence of elderly patients, especially should they lack fundamental training in the management of dementia and Alzheimer’s. Patients with other kinds of mental disorders may have particular psychosocial and psychological stressors activated in the clinical setting. Some may be affected by positive psychotic symptoms and have reduced impulse control, often not understanding the process of care-giving well. Patients’ legal status in being admitted to psychiatric units, ethnicity, time in hospital, diagnosis, and drug compliance may all count as factors linked to WPV [37].

Our findings highlight the extent of the violence suffered by Macau healthcare professionals at the hands of patients and their visitors. A 12-month prevalence rate of 57.2% of professionals affected by violence points to a significant public health issue.

### 4.6. Worry about WPV

With the exception of sexual and racial harassment, worry about WPV turned out to be a significant correlate for all other kinds of abuse considered in this study. Verbal abuse and bullying have higher prevalence estimates than physical assault, yet professionals’ concern about WPV consistently emerges as a significant correlate in physical assault, verbal abuse, bullying, and sexual harassment. It would seem that victims know what is coming in terms of the safety or broader tenor of the atmosphere in which they operate. The implication is that non-physical forms of abuse should not be considered as less serious than physical assault. The high prevalence rate for violence, per se, suggested the lack of any formal violence prevention program being in place [38], disturbingly suggesting a cultural acceptance of violence, including a lack of administrative support to address WPV within healthcare settings [39].

Worrying about WPV has not been historically unfounded, in the sense that nurses are arguably one of the most vulnerable occupational groups in their exposure to WPV [19]. Nurses’ position below doctors in the medical hierarchy, and the low degree of autonomy with which they go about their work, further explains their vulnerability to patient violence [40]. Research has also highlighted the impact of negative affectivity (NA) as a potential antecedent of WPV. This term refers to an individual’s level of pervasively feeling bad and thinking badly of themselves [41]. Individuals with NA may perceive others’ behavior as more personally critical than it is [42], leading to disproportionate distress in interpersonal conflict.

Nevertheless, it is not unusual for individuals to experience anxiety or fear after being physically assaulted, abused, or bullied by patients or visitors. Anxious doctors and nurses may find it harder to work productively and communicate effectively with patients [15]. In these situations, the stress-response system becomes dysregulated as professionals go into hyper-arousal [43]. The relationship between staff anxiety and patient aggression is complex. Anxious healthcare providers may avoid interacting with patients, leading to their being misinterpreted; equally, providers’ lower tolerance of minor forms of patient disruptiveness may eventually heighten the risk of violence in healthcare settings. In this regard, it can be argued that staff anxiety and the hyper-arousal effect tie in closely with WPV.

## 5. Recommendations

We would recommend for healthcare professionals of all ranks to receive training on a mandatory basis in de-escalation skills [36] and in breakaway techniques to avert the risk to themselves of both physical and psychological harm. Fernandes et al. [37] show how educational training programs can help nurses prevent aggressive behavior developing into violence, as they become skilled in early intervention. Healthcare providers should make managing violence part of the continuing education of newly-recruited employees, particularly frontline healthcare workers.

The Macau Health Authority should consider framing a zero-tolerance policy for WPV enforced in collaboration with the police. All employees should be assured that no one reporting abuse could face reprisals—a circumstance that could prove instrumental in eliminating WPV [36]. In 2013, seven US states enacted laws to reduce WPV against healthcare workers, putting WPV prevention programs in place. Health policy-makers in Macau should follow their lead and think about expanding WPV prevention programs to all community day centers, and home health [44] and out-patient clinics [45,46]. To sound a more cautionary note, a recent systematic review of the efficacy managing aggression by improving training for nurses in six countries (the USA, United Kingdom, Canada, Australia, Switzerland, and Germany) (380 cases) concluded no significant differences flowed from pre-training in dealing with violent incidents [47]. This illustrates the need to engage with WPV not through throwing responsibility onto individuals, but at an organizational level.

## 6. Limitations

The study shares the feature of cross-sectional convenience samples of possibly being liable to self-selection bias—respondents who had been subject to WPV may have been more likely to answer the survey. Our respondents do seem to have been representative of the study population. Approximately 1 in 15 physicians participated, and 1 in 3 nurses. Those not replying might not have experienced WPV. There is a contrary possibility, that the victims of WPV would have been disinclined to reply as they did not want to relive their negative experiences [15].

## 7. Conclusions

WPV is detrimental to individual’s physical, psychological and mental well-being. WPV seems to be a significant occupational hazard for physicians and nurses in Macau. Nonetheless, patients’ violence or aggressive behaviors should be regarded as a preventable occupational risk. The health authority in Macau should have a raft of health policy and training programs specific to managing violence to equip its frontline healthcare workers to deal with aggressive episodes in clinical settings. Even so, training may not be sufficient to avert violence without further organizational support in the form of reporting and monitoring measures. The authority should set up WPV prevention initiatives at organizational level allowing hospitals and other institutions to intervene to mitigate the negative impact of violence on healthcare workers. Physicians and nurses who are at disproportionately high risk of WPV should strengthen their stress-coping strategies and foster their level of resilience to minimize the negative psychological consequences of violence that jeopardize their psychological and mental wellbeing.

## Figures and Tables

**Table 1:** Frequency distribution of respondents by socio-demographic characteristics and selected variables (*n* = 720).

Variables	*n* (%)
**Sex**	
Male	153 (21.3)
Female	567 (78.8)
**Age (years)**	
19–24	85 (11.8)
29–34	339 (47.1)
35–44	149 (20.7)
45–54	103 (14.3)
55–60+ ^a^	44 (6.1)
**Marital Status**	
Single	293 (40.7)
Married/cohabitant	412 (57.2)
Widowed/separated/divorced ^a^	15 (2.1)
**Profession**	
Physician ^a^	107 (14.9)
Nurses	613 (85.1)
**Rank**	
Managers ^a^	20 (2.8)
Frontline staff	700 (97.2)
**Work Experience (years)**	
0–10	449 (62.4)
11–20+ ^a^	271 (37.6)
**Employment Sector**	
Public	492 (68.3)
Private ^a^	228 (31.7)
Shift Work ^#^	544 (75.6)
Night Duty (18:00–07:00) ^#^	509 (70.7)
Direct Patient Care ^#^	693 (96.3)
Direct Patient Care (Newborn) ^#^	106 (15.4)
Direct Patient Care (Infants) ^#^	121 (17.5)
Direct Patient Care (Children) ^#^	159 (23.0)
Direct Patient Care (Adolescents) ^#^	233 (33.8)
Direct Patient Care (Adults) ^#^	617 (89.4)
Direct Patient Care (Elderly) ^#^	493 (71.4)
50% Direct Patient Care (Physical Disability) ^#^	132 (19.2)
50% Direct Patient Care (Intellectual Disability) ^#^	51 (7.4)
50% Direct Patient Care (Home Care) ^#^	79 (11.5)
50% Direct Patient Care (Terminal Illness) ^#^	153 (22.3)
50% Direct Patient Care (HIV) ^#^	19 (2.8)
50% Direct Patient Care (Psychiatric Disorder) ^#^	101 (14.7)
50% Direct Patient Care (Maternity) ^#^	105 (15.3)
50% Direct Patient Care (Elderly) ^#^	438 (63.8)
50% Direct Patient Care (Occupational Health) ^#^	85 (12.4)
50% Direct Patient Care (School Hygiene) ^#^	42 (6.1)
Worry about WPV ^#^	630 (36.8)

^a^ Reference category; ^#^ Without.

**Table 2:** Prevalence of physical assault, verbal abuse, bullying, sexual harassment, and racial harassment towards physicians and nurses in Macau (*n* = 720).

Profession	Physical Assault (*n*, %)	Verbal Abuse (*n*, %)	Bullying (*n*, %)	Sexual Harassment (*n*, %)	Racial Harassment (*n*, %)
**Physician**	4 (3.7)	41 (38.3)	13 (12.1)	4 (3.7)	4 (3.7)
**Nurse**	111 (18.1)	343 (56.0)	89 (14.5)	29 (4.7)	15 (2.4)
**x^2^**	4.02 *p* < 0.001 ***	11.39 *p* < 0.001 ***	0.42 *p* = 0.52 **	0.21 *p* = 0.65 **	0.59 *p* = 0.44 **

** *p* value significant at 0.01 (2-tailed); *** *p* value significant at <0.001 (2-tailed).

**Table 3:** Frequency (%) and Pearson χ^2^ of perpetrators towards physicians and nurses in relation to physical assault, verbal abuse, bullying, sexual harassment, and racial harassment (*n* = 720).

Perpetrator	Physical Assault (*n*, %)		χ^2^	Verbal Abuse (*n*, %)		χ^2^	Bullying (*n*, %)		χ^2^	Sexual Harassment (*n*, %)		χ^2^	Racial Harassment (*n*, %)		χ^2^
	Physician	Nurse		Physician	Nurse		Physician	Nurse		Physician	Nurse		Physician	Nurse	
Patient	1 (1)	100 (99)	15.30 ***	25 (10.9)	204 (89.1)	0.03	7 (12.7)	48 (87.3)	0.08	4 (21.1)	15 (78.9)	3.35	1 (14.3)	6 (85.7)	0.31
Patient’s family members	3 (23.1)	10 (76.9)	16.77 ***	14 (10.1)	125 (89.9)	0.08	5 (20.0)	20 (80.0)	2.09	1 (20.0)	4 (80.0)	0.34	2 (33.3)	4 (66.7)	0.80
Colleagues	0 (0)	2 (100)	0.07	0 (0)	34 (100)	4.46 *	0 (0)	18 (100)	2.95	0 (0)	9 (100)	1.71	1 (16.7)	5 (83.3)	0.10

* *p* < 0.05; *** *p* < 0.001; χ^2^: Pearson Chi-square.

**Table 4:** Binary logistic regression analysis between physical assault, verbal abuse, bullying, sexual harassment, racial harassment, and other socio-demographic characteristics.

Variability	Physical Assault			Verbal Abuse			Bullying			Sexual Harassment			Racial Harassment		
	cOR	95% CI		cOR	95% CI		cOR	95% CI		cOR	95% CI		cOR	95% CI	
		Upper Bound	Lower Bound		Upper Bound	Lower Bound		Upper Bound	Lower Bound		Upper Bound	Lower Bound		Upper Bound	Lower Bound
**Sex**															
Male ^#^	-	-	-	-	-	-	-	-	-	-	-	-	-	-	-
Female	1.16	0.70	1.92	1.13	0.79	1.62	0.98	0.59	1.63	1.23	0.50	3.02	1.01	0.33	3.10
**Age**															
19–24	3.17	0.87	11.54	1.97	0.93	4.16	0.59	0.17	2.06	2.12	0.23	19.60	0.25	0.02	2.84
25–34	3.17	0.95	10.55	2.66	1.39	5.11	1.51	0.57	4.00	1.99	0.26	15.45	0.44	0.09	2.20
35–44	2.37	0.67	8.32	1.64	0.82	3.27	1.07	0.37	3.07	3.09	0.39	24.86	0.58	0.10	3.27
44–54	1.65	0.44	6.24	1.47	0.71	3.04	1.65	0.57	4.77	1.29	0.13	12.75	1.07	0.20	5.74
55–60+ ^#^	-	-	-	-	-	-	-	-	-	-	-	-	-	-	-
**Marital Status**															
Single	0.47	0.16	1.51	1.20	0.43	3.40	0.42	0.13	1.39	0.86	0.11	6.95	0.09 **	0.02	0.54
Married/Cohabitant	0.29 *	0.10	0.89	0.88	0.32	2.48	0.46	0.14	1.49	0.53	0.07	4.29	0.21	0.04	1.04
Widowed/Separated/Divorced ^#^	0.01	-	-	0.14	-	-	0.37	-	-	0.38	-	-	0.03	-	-
**Profession**															
Physician ^#^	-	-	-	-	-	-	-	-	-	-	-	-	-	-	-
Nurses	5.62 **	2.03	1.58	2.05 **	1.35	3.13	1.23	0.66	2.29	1.28	0.44	3.71	0.65	0.21	1.99
**Rank**															
Managers ^#^	-	-	-	-	-	-	-	-	-	-	-	-	-	-	-
Frontline	1.09	0.31	3.78	2.17	0.86	5.52	1.50	0.34	6.56	0.91	0.12	7.01	45,071,981.9	0.000	-
**Work experience**															
0–10 years	1.56 *	1.01	2.40	1.60 **	1.18	2.17	0.88	0.58	1.35	1.41	0.66	3.01	0.43	0.17	1.08
11–20+ ^#^	-	-	-	-	-	-	-	-	-	-	-	-	-	-	-
**Employment Sector**															
Public	1.79 *	1.11	2.87	1.50 *	1.10	2.06	2.59 **	1.50	4.48	0.92	0.44	1.94	1.76	0.58	5.37
Private ^#^	-	-	-	-	-	-	-	-	-	-	-	-	-	-	-
Shift Work ^a^	3.56 ***	1.86	6.79	2.36 ***	1.66	3.34	1.29	0.77	2.16	1.21	0.52	2.84	0.55	0.21	1.41
Night Duty ^a^ (18:00–07:00)	2.26 **	1.34	3.81	2.16 ***	1.56	3.01	1.37	0.84	2.24	1.08	0.50	2.37	1.53	0.50	4.68
Direct Patient Care ^a^	4.53	2.15	9.52	1.54 *	1.08	2.20	0.92	0.56	1.51	4.67 *	1.10	19.77	2.11	0.48	9.39
Direct Patient Care (Newborn) ^a^	0.43 *	0.21	0.88	0.69	0.46	1.05	0.65	0.34	1.27	0.56	0.17	1.86	2.58	0.88	7.58
Direct Patient Care (Infants) ^a^	0.31 *	0.15	0.66	0.81	0.54	1.19	0.67	0.36	1.26	0.66	0.23	1.92	2.92 *	1.24	8.18
Direct Patient Care (Children) ^a^	0.39 *	0.21	0.71	0.65 *	0.45	0.92	0.77	0.45	1.31	0.76	0.31	1.89	2.04	0.73	5.71
Direct Patient Care (Adolescent) ^a^	0.92	0.60	1.42	1.23	0.89	1.69	1.62 *	1.05	2.50	1.36	0.66	2.81	2.00	0.74	5.39
Direct Patient Care (Adults) ^a^	0.99	0.52	1.91	1.45	0.89	2.36	3.14 *	1.12	8.81	0.10	88,367,890.3	0.000	0.82	0.18	3.70
Direct Patient Care (Elderly) ^a^	5.85 ***	2.72	11.09	1.99 ***	1.43	2.79	1.69 *	1.00	2.84	1.77	0.72	4.37	6.15	0.81	46.88
50% Direct Patient Care (Physical Disability) ^a^	2.62 ***	1.68	4.09	2.28 ***	1.52	3.44	2.33 ***	1.45	3.74	2.31 *	1.09	4.92	1.13	0.37	3.45
50% Direct Patient Care (Intellectual Disability) ^a^	0.72 **	1.47	5.07	4.21 ***	2.01	8.79	3.05 ***	1.61	5.75	1.85	0.62	5.49	3.52*	1.12	11.04
50% Direct Patient Care (Homecare) ^a^	0.89	0.46	1.70	0.88	0.55	1.40	1.61	0.89	2.91	2.27	0.95	5.43	1.46	0.42	5.13
50% Direct Patient Care (Terminal Illness) ^a^	1.42	0.90	2.24	1.89 ***	1.30	2.76	1.55	0.96	2.50	1.39	0.63	3.07	0.93	0.30	2.84
50% Direct Patient Care (HIV) ^a^	1.80	0.64	5.10	1.82	0.68	4.83	2.85 *	1.06	7.69	2.50	0.55	11.33	4.51	0.96	21.06
50% Direct Patient Care (Psychiatric Disorder) ^a^	2.79 ***	1.73	4.50	4.27 ***	2.53	7.22	3.82 ***	2.35	6.23	2.82 *	1.29	6.14	1.09	0.31	3.81
50% Direct Patient Care (Maternity) ^a^	0.36 **	0.17	0.77	0.49 ***	0.27	0.63	0.26 **	0.10	0.66	0.56	0.17	1.88	0.65	0.15	2.84
50 % Direct Patient Care (Elderly) ^a^	2.98 ***	1.80	4.92	1.92 ***	1.40	2.63	1.53	0.96	2.44	0.82	0.40	1.70	0.78	0.31	1.96
50% Direct Patient Care (Occupational Health) ^a^	0.80	0.42	1.53	1.14	0.72	1.81	0.97	0.51	1.87	1.68	0.67	4.22	2.63	0.92	7.48
50% Direct Patient Care (School Hygiene)	0.11	0.02	0.83	0.60	0.32	1.13	0.61	0.21	1.75	1.03	0.24	4.44	1.85	0.41	8.28
Worry about WPV	6.25 **	1.94	20.11	5.17 ***	3.04	8.78	3.96 **	1.42	11.04	2.28	0.54	9.68	0.39	0.14	1.10

* *p* < 0.05; ** *p* < 0.01; *** *p* < 0.001. cOR: cumulative odds ratios. 95% CI: 95% confidence interval. ^#^ reference category. ^a^ without.

**Table 5:** Multivariate logistic regression model predicting physical assault, verbal abuse, bullying, sexual harassment, and racial harassment towards physicians and nurses in Macau.

Variables	β	SE	AOR (95% CI)
**Physical Assault**			
Constant	−5.245	0.84	-
Shift Duty ^#^	0.906	0.38	2.48 (1.18, 5.20) *
Direct Patient Care (Children) ^#^	−1.015	0.33	0.36 (0.19, 0.69) **
Direct Patient Care (Elderly) ^#^	1.724	0.37	5.61 (2.71, 11.60) ***
50% Direct Patient Care (Physical Disability) ^#^	0.515	0.25	1.67 (1.02, 2.74) *
50% Direct Patient Care (Psychiatric Disorder) ^#^	0.654	0.27	1.92 (1.13, 3.27) **
Worry About WPV ^#^	1.506	0.74	4.51 (1.06, 19.17) *
**Verbal Abuse**			
Constant	−2.085	0.35	-
Work Experience (0-10 years) ^a^	0.380	0.18	1.46 (1.03, 2.07) *
Night Duty (18:00–07:00) ^#^	0.658	0.19	1.93 (1.34, 2.79) ***
Direct Patient Care (Elderly) ^#^	0.478	0.19	1.61 (1.10, 2.36) **
50% Direct Patient Care (Psychiatric Disorder) ^#^	1.169	0.28	3.22 (1.87, 5.55) ***
50% Direct Patient Care (Maternity) ^#^	−0.595	0.24	0.55 (0.34, 0.89) *
Worry about WPV ^#^	1.362	0.30	3.90 (2.17, 7.02) ***
**Bullying**			
Constant	−5.245	0.84	-
Shift Duty ^#^	0.906	0.38	2.48 (1.18, 5.20) *
Direct Patient Care (Children) ^#^	−1.015	0.33	0.36 (0.19, 0.69) **
Direct Patient Care (Elderly) ^#^	1.724	5.61	5.61 (2.71, 11.60) ***
50% Direct Patient Care (Physical Disability) ^#^	0.515	0.25	1.67 (1.02, 2.74) *
50% Direct Patient Care (Psychiatric Disorder) ^#^	0.654	0.27	1.92 (1.13, 3.27) *
Worry about WPV ^#^	1.506	0.74	4.51 (1.06, 19.17) *
**Sexual Harassment**			
Constant	−4.713	0.74	-
Direct Patient Care ^#^	1.521	0.74	4.58 (1.07, 19.65) *
Direct Patient Care (Home Care) ^#^	1.045	0.46	2.84 (1.16, 7.00) *
Direct Patient Care (Psychiatric Disorder) ^#^	0.989	0.41	2.69 (1.21, 5.95) *
**Racial Harassment**			
Constant	−4.164	0.35	-
50% Direct Patient Care (Physical Disability) ^#^	1.384	0.60	3.99 (1.22, 13.03) *
Direct Patient Care (Infants) ^#^	1.031	0.53	2.80 (0.99, 7.97) *

* *p* < 0.05; ** *p* < 0.01; *** *p* < 0.001. AOR: adjusted odds ratio. 95% CI: 95% Confidence Interval. SE: Standard error. ^a^ 11–20 years; ^#^ without.
